# Chitosan-Based Biopolymer Films for Sustainable Functional Integration

**DOI:** 10.3390/ma19153315

**Published:** 2026-08-04

**Authors:** Kiril Dimitrov, Imasha Danwatte, Vesela Stoycheva, Leonid M. Goldenberg, Daniel Pinkal, Michael Wegener, Christian Dreyer, Michael Herzog

**Affiliations:** 1 Fiber Composite Material Technologies Faculty of Engineering and Natural Sciences, Technical University of Applied Sciences Wildau, Hochschulring 1, 15745 Wildau, Germany; goldenberg@th-wildau.de; 2 High Performance Polymers, Faculty of Engineering and Natural Sciences, Technical University of Applied Sciences Wildau, Hochschulring 1, 15745 Wildau, Germany; imasha.danwatte2@gmail.com (I.D.); vesela.stoycheva@th-wildau.de (V.S.); michael.herzog@th-wildau.de (M.H.); 3 Fraunhofer-Institute for Applied Polymer Research, Research Division Polymeric Materials and Composites PYCO, Schmiedestr. 5, 15745 Wildau, Germany; 4 Fraunhofer-Institute for Applied Polymer Research, Research Division Functional Polymer Systems, Geiselbergstr. 69, 14476 Potsdam, Germany; daniel.pinkal@iap.fraunhofer.de (D.P.); michael.wegener@iap.fraunhofer.de (M.W.)

**Keywords:** chitosan films, biopolymer films, electromechanical activity, composite materials, sensor functionality, rheology, TGA, thermo-optic coefficient, FTIR

## Abstract

The objective of this study was to identify chitosan formulations suitable for sustainable functional integration into composite materials. To this end, the influence of solvent type (acetic acid and lactic acid) and chitosan molecular weight on film formation, rheological behavior, thermal response, thermo-optical properties, chemical structure, and piezoelectric performance was systematically investigated. Optimized casting and drying procedures produced transparent, homogeneous, and mechanically stable films suitable for comprehensive characterization. Rheological and thermo-optical analyses demonstrated that solvent selection strongly influenced polymer network formation and molecular mobility. Films prepared using acetic acid exhibited denser and stiffer polymer networks with improved dimensional stability, whereas lactic acid produced more flexible and elastic films. Thermogravimetric analysis revealed only minor differences in the intrinsic thermal stability of the investigated films, while FTIR confirmed that the solvent systems did not alter the chemical structure of chitosan. Electrical measurements carried out whilst the system was subjected to periodic mechanical excitation revealed weak but equally periodic electrical signals, which demonstrate a sensor functionality. These results demonstrate that chitosan films possess tunable structural, thermal, and potential mechanoelectrical sensor properties and highlight their potential as sustainable, functionally integrated components in composite material systems.

## 1. Introduction

As environmental control systems become increasingly important in agriculture, the need for sophisticated and sustainable sensor technology is growing. Thanks to the latest technological developments, such sensors can be integrated into easily moldable materials (e.g., composites) and adapted to cultivation conditions. Functional integration into composite materials is an important and essential concept for making processes, components, and systems more cost-effective, resource-efficient, lighter, and more reliable. A decisive disadvantage of functional integration, however, is that components manufactured in this way inevitably consist of multimaterial combinations, which significantly complicates their recycling and material recovery. Today, sensors are increasingly integrated into highly loaded components to improve the safety, reliability, and functionality of systems, vehicles, machines, and structures. Pressure sensors based, e.g., on piezoelectric active materials are emerging as a key technology for monitoring environmental and mechanical conditions in agricultural systems and other engineering applications. 

Piezoelectric materials are able to convert mechanical energy into electrical energy and vice versa [1]. Mechanical deformation generates an electrical signal that can be processed and analyzed, while an applied electric field induces strain, enabling controlled motion. This effect makes these materials excellent candidates for sensing applications and for integration into various types of structures from different materials, such as shoe soles [2]. They are also promising materials for medical applications, and piezoelectric materials with biodegradable properties are being actively explored [3]. There is a huge number of publications describing the use of polyvinylidene fluoride (PVDF) as a piezoelectric material for a wide range of applications, including sensors for biomedical monitoring (e.g., pulse and respiration), robotics (tactile and pressure sensing), and structural health monitoring [4,5,6,7]. Owing to its high β-phase content, PVDF efficiently converts mechanical stress, vibrations, and acoustic signals into electrical signals [8]. However, this material also has drawbacks, such as possible toxicity arising from thermal degradation [9]. Therefore, there is a growing demand for a new generation of bio-based, non-toxic piezoelectric or other electromechanical active polymers that combine simple processing routes with favorable mechanical and thermal properties.

Polysaccharides, especially chitin and chitosan, are promising alternative materials. These biopolymers are of particular importance due to their high natural abundance and relatively low cost of fabrication. In addition, the materials are renewable, biodegradable, and generally biocompatible, which makes them attractive candidates for the development of sustainable, bio-based piezoelectric polymers [10]. Chitin is widely distributed in nature and occurs in the exoskeletons of arthropods (such as crustaceans and insects), the skeletal components of marine zooplankton, the cell walls of fungi and yeast, and the tubes of pogonophora (see worms living in tubes) [11,12]. It is also present in the walls of ciliate cysts and diatom spines [13,14]. A deacetylation process of chitin is required for the production of chitosan. At present, chitin and chitosan are primarily sourced from arthropods, particularly crustaceans [15]. The most common raw materials for industrial chitosan production include crabs, prawns, and lobsters [16].

The piezoelectric effect originates from the non-centrosymmetric arrangement of molecules within a material [17]. Although chitin and chitosan are semi-crystalline polymers with partially ordered molecular structures [18], crystallinity alone does not guarantee a strong piezoelectric response. Effective piezoelectricity requires a non-centrosymmetric arrangement of dipoles and a sufficient degree of molecular orientation within the material [19]. Consequently, various strategies have been proposed to enhance the piezoelectric performance of chitosan-based films. These include the incorporation of chitosan nanocrystals into polymer matrices [20], the fabrication of chitosan nanofibers via electrospinning [21], and the addition of functional fillers to chitosan [22].

Not only the sensor response but also the thermomechanical properties of the investigated materials are of particular relevance for the present work. In this context, thermo-optical measurements represent a reliable method for determining phase transitions, such as glass transition and melting behavior, in thin-film materials. These transitions are often difficult to characterize using conventional techniques such as differential scanning calorimetry (DSC) or dynamic mechanical analysis (DMA), which typically require larger sample volumes or bulk specimens [23].

A thorough understanding of these thermomechanical properties is essential for assessing the processability of the materials and their suitability for integration into polymer-based composite systems. Therefore, the compatibility of the investigated materials with polymer matrices and their potential for subsequent functional integration constitute an important aspect of this study. Such integration concepts are not limited to the materials examined here but may also be extended to other functional material systems, including photonic components fabricated from transparent materials [24].

## 2. Experimental

### 2.1. Materials

Low-molecular-weight chitosan (50,000–190,000 Da, 75–85% degree of deacetylation), medium-molecular-weight chitosan (190,000–310,000 Da, 75–85% degree of deacetylation), and high-molecular-weight chitosan (310,000–375,000 Da, degree of deacetylation >75%) (all Sigma-Aldrich (Merck), Taufkirchen, Germany, practical grade) were used as received without further purification. Deionized water was used for all solution preparations. Acetic acid (100%, Ph. Eur. grade) was used for the preparation of aqueous acetic acid solutions and employed as a solvent for chitosan dissolution. Lactic acid (Sigma-Aldrich, analytical grade) was used without further purification as an alternative solvent owing to its bio-based origin, low toxicity, and effectiveness in promoting chitosan solubility.

### 2.2. Film Preparation and Properties

Aqueous acid solutions of acetic acid (AcOH) and lactic acid (LA) (1v%) were prepared using deionized water with a total volume of 100 mL. Chitosan (1.0 g) was added to each solution to obtain a concentration of 1% (*w*/*v*). The mixtures were stirred at 80 °C at 700 rpm for 24 h to ensure dissolution. Following dissolution, the solutions were filtered to remove undissolved particles. Incomplete solubility, particularly for high-molecular-weight chitosan, was observed. This may be attributed to its lower degree of deacetylation and increased chain entanglement. The amount of undissolved material retained during filtration was not quantified; therefore, a slight reduction in the effective polymer concentration and a potential shift in the molecular weight distribution of the casting solution cannot be excluded. However, the filtration step was intentionally employed to ensure that the casting solutions were free of undissolved particles, thereby producing homogeneous, defect-free films. The presence of residual insoluble particles could act as structural defects or stress concentration sites and interfere with the rheological, mechanical, and electromechanical characterization of the films. By removing these particles, the influence of undissolved material on the measured film properties was minimized. Furthermore, all formulations were prepared using the same dissolution and filtration protocol, ensuring consistent sample preparation and enabling a reliable comparative evaluation of the different chitosan grades.

The prepared chitosan films exhibited thicknesses ranging from 0.023 to 0.033 mm (Table 1). For the AcOH-based films, the film thickness generally increased with increasing chitosan molecular weight. This observation may indicate increased retention of residual lactic acid and/or bound water within the film structure. In contrast, AcOH-based films exhibited lower final weights, suggesting more efficient solvent removal during drying and the formation of a denser polymer network.

**Figure 1:**
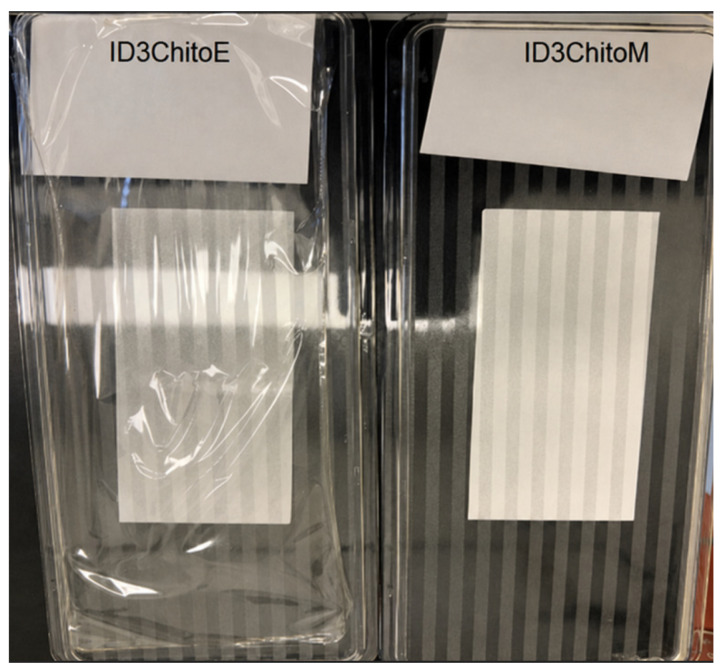
Chitosan films prepared from (AcOH, **left**) and (LA, **right**) solutions in PET plates.

The molecular weight of chitosan significantly influenced its dissolution behavior in both solvent systems. Increasing molecular weight increased solution viscosity owing to longer polymer chains and greater chain entanglement, thereby hindering complete dissolution. Low-molecular-weight chitosan (ID1) dissolved rapidly, producing brittle films in acetic acid (AcOH) but highly elastic films in lactic acid (LA). Medium-molecular-weight chitosan (ID2) exhibited the best processability, dissolving readily in both solvent systems without filtration and yielding homogeneous, defect-free films. In contrast, high-molecular-weight chitosan (ID3) dissolved more slowly and required filtration before film casting because of undissolved particles. Nevertheless, the resulting films remained transparent and mechanically stable. The solutions were cast onto polyethylene terephthalate (PET) substrates and dried at 30 °C for 72 h (Figure 1). 

**Figure 2:**
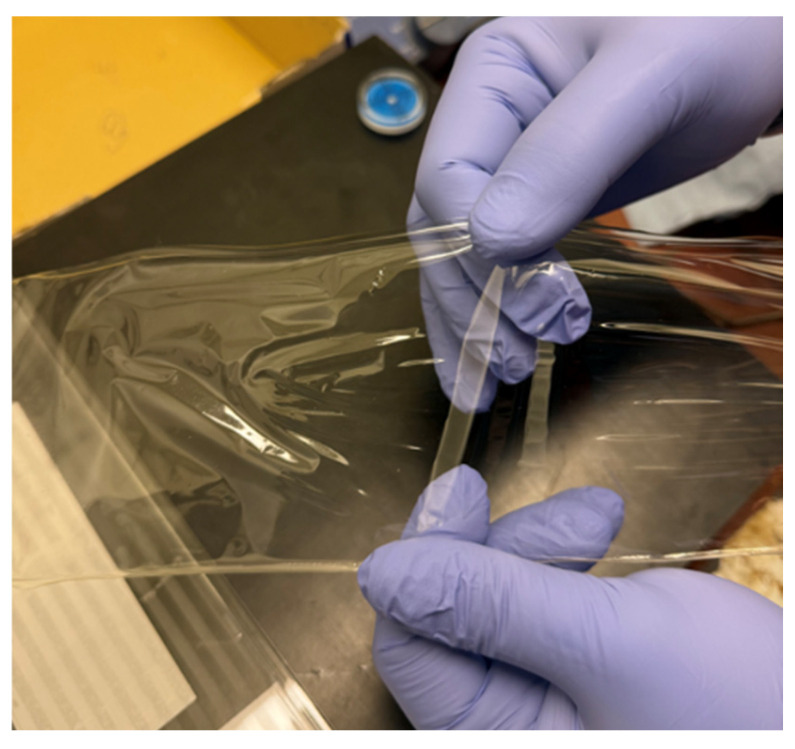
Chitosan film made from AcOH (ID2ChitoE).

**Figure 3:**
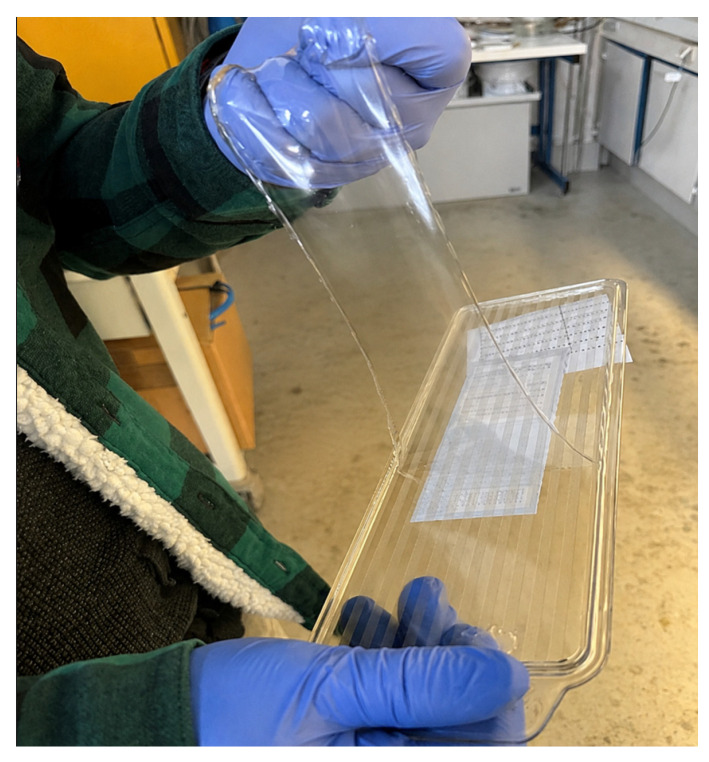
Chitosan film made from LA (ID2ChitoM).

Figure 2 and Figure 3 show the dried films prepared from medium-molecular-weight chitosan (ID2) using AcOH and LA, respectively. Both solvent systems produced transparent, homogeneous films without visible defects. These observations indicate that AcOH promotes the formation of a denser and more rigid polymer network, whereas LA enhances polymer chain mobility through stronger polymer–solvent interactions. 

### 2.3. Methods

#### 2.3.1. Preparation of Samples for Sputtering

**Figure 4:**
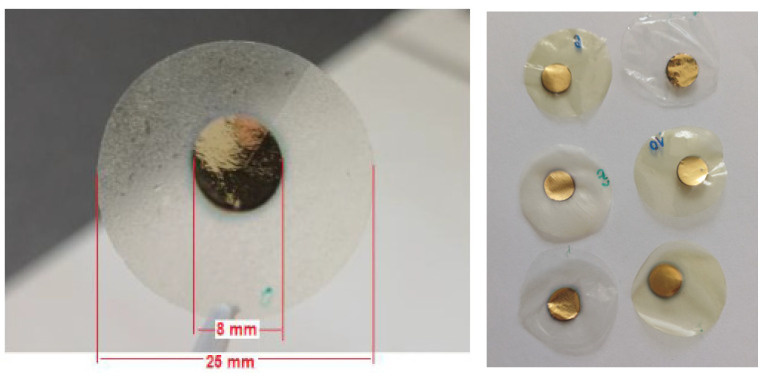
Chitosan films sputtered with a gold electrode.

The thickness of the films was measured to ensure consistency and comparability of results prior to sputtering. Measurements were performed using a precision thickness gauge with an operating range between 0 and 25 mm and a precision of 0.001 mm. The film thickness was critical for evaluating the uniformity of metal deposition and the accuracy of subsequent mechanoelectric measurements. Gold electrode layers were deposited onto the prepared chitosan films using a Leica M SCD500 (Leica Microsystems, Vienna, Austria) high-vacuum sputter coater operated with an argon plasma. The system enables uniform coating with minimal thermal stress, making it ideal for sensitive materials. Gold electrodes were used in this study. A 20 nm chromium adhesion layer was first sputter-deposited at 100 mA, followed by the deposition of a 100 nm gold layer at 50 mA under the same vacuum and gas conditions (Figure 4). 

#### 2.3.2. Fourier-Transform Infrared Spectroscopy Analysis

Fourier-transform infrared (FTIR) spectroscopy was employed to characterize the chemical structure of the chitosan films. Spectra were recorded using a Varian 620-IR FTIR system (Varian, Inc., Palo Alto, CA, USA) in the spectral range of 400–4000 cm^−1^. The obtained spectra were used to identify characteristic functional groups and to evaluate possible molecular interactions within the films.

#### 2.3.3. Rheological Analysis

Rheological analysis was conducted using an Anton Paar Physica MCR 301 (Anton Paar GmbH, Graz, Austria) rotational rheometer fitted with a 50 mm plate–plate geometry. The time-dependent viscoelastic behavior of chitosan solutions was evaluated using oscillatory time-sweep tests. In order to maintain thermal stability, a Julabo AWC100 (JULABO GmbH, Seelbach, Germany) air-water recirculating cooler was used to support a Peltier-controlled system that maintained the temperature with an accuracy of ±0.01 °C. The rheometer can measure across a broad frequency spectrum from 10^−5^ to 10^2^ Hz and temperatures from −40 to 200 °C. However, only ambient conditions were used in this investigation. Insight into the curing and structural development of the polymer solutions during solvent evaporation and network creation was provided by the applied setup, which made it possible to monitor polymer network development during solvent evaporation, G′, and G″.

#### 2.3.4. Thermogravimetric Analysis

This investigation was performed using a TGA/SDTA 851 instrument (Mettler-Toledo, Schwerzenbach, Switzerland). Samples were heated from 25 °C to 850 °C at a heating rate of 10 K min^−1^ under a nitrogen atmosphere using alumina crucibles. The mass loss as a function of temperature was recorded to evaluate the thermal stability and degradation behavior of the biopolymer materials.

#### 2.3.5. Optical Characterization—Thermo-Optic Coefficient

The films were dried in a vacuum oven at 70 °C for 2 days before these measurements. The optical properties of film samples were measured by m-line spectroscopy using a Metricon 2010/M Prism Coupler System (Metricon Corporation, Pennington, NJ, USA) in a temperature range up to 200 °C at 1547 nm wavelength. The films are pressed onto silicon wafers or used as free-standing films pressed directly onto the coupling prism. In this technique, the sample is brought into contact with the base of a prism by applying pressure. A laser beam strikes the base of the prism and is totally reflected onto a photodetector. By rotating the prism, at certain values of the incident angle, the photons can tunnel across the air gap between the prism surface and the sample into the film and enter into a guided mode, causing a sharp drop in the intensity of light reaching the photodetector (called mode, deep in the curve). The angular location of the first mode determines the film index, while the angular difference between the modes determines the thickness. Such behavior (single-film mode) was usually observed when the film was pressed onto a Si waver. In a bulk/substrate mode (for thick films where the modes cannot be observed), the refractive index is calculated at the position of the critical angle knee. The thermo-optic coefficient (TOC) was evaluated from temperature-dependent refractive index measurements (with a step of 10 °C) using a temperature-regulated optical prism. The TOC was calculated as the slope of the refractive index–temperature dependence. Changes in the TOC or deviations from linear behavior were used to identify thermal transitions, including glass transition and melting processes. Furthermore, discontinuities in the refractive index–temperature relationship may indicate additional thermally activated phenomena, such as the removal of residual solvent or absorbed water. Typically, two consecutive measurement runs were carried out at the same location to ensure reproducibility.

#### 2.3.6. Characterization of Sensor Function and Electrical Pretreatment

To investigate the sensor functionality under mechanical excitation, the samples were subjected to a fixed pre-load force of 8 N superimposed by a sinusoidal dynamic force modulated at 2 Hz. The electrical charge displacement in response to the applied dynamic force amplitudes (peak-to-peak) of about 0.5 N, 1 N, 2 N and 4 N was measured by a charge amplifier connected to a digital multimeter. The force signal was measured simultaneously by a load cell connected to a load cell amplifier and another digital multimeter. The corresponding peak-to-peak values of force (Fpp) and charge (Qpp) were derived from the signals. A sensor activity can be calculated by dividing Qpp by Fpp. 

Some samples were electrically pretreated in order to evaluate changes in sensor functionality. For this purpose, a triangular electric field with a frequency of 1 kHz and stepwise increasing amplitudes up to, e.g., 20 or 60 MV/m was applied by means of electrical measurement and poling equipment aixACCT (Aachen, Germany), type TF Analyzer 2000E, combined with a high voltage amplifier TREK 10/40A-HS, (Lockport, USA).

## 3. Results and Discussion

The fundamental aim of this study is to prepare biopolymeric materials based on chitosan with a mechanoelectrical sensor functionality. This section is an assimilation of experimental data obtained from several methodologies such as rheology (oscillatory and viscometry), TGA, TOC and FTIR. Furthermore, the sensor function of the developed films was evaluated. This section is intended to highlight the physical, thermal, morphological, and rheological attributes of chitosan films and to discuss the possibilities and limitations of their future functional integration in composite materials.

### 3.1. Rheological Analysis

#### Dynamic Rheology

This investigation was employed to study the time-dependent rheological behavior of chitosan solutions during drying and hardening. The measurements provided insight into the evolution of the viscoelastic properties and the transition from a liquid-like to a solid-like state as solvent evaporation progressed. The tests were conducted at a constant temperature of 25 °C, a constant frequency of 1 Hz and an applied strain amplitude of 10% in order to replicate the real-state behavior of the materials under conditions of slight mechanical stress. No solvent trap, humidity control, or edge sealing were employed because solvent evaporation was an inherent part of the investigated process. Consequently, the increase in the storage modulus (G′) and the crossover of the storage modulus (G′) and loss modulus (G″) reflect the combined effects of solvent evaporation, increasing polymer concentration, chain entanglement, and polymer network formation during drying. Therefore, the observed crossover times are used as comparative indicators of the relative rates of network development and film hardening rather than as absolute intrinsic gelation times.

Freshly prepared chitosan solutions in either LA or AcOH were analyzed immediately after preparation. The evolution of storage modulus (*G*′) and loss modulus (*G*″) over time was recorded for each sample. At the beginning of the measurements, *G*″ exceeded *G*′, indicating predominantly viscous (liquid-like) behavior. Accordingly, *G*″ is shown as the upper curve and *G*′ as the lower curve in the graphs.

**Figure 5:**
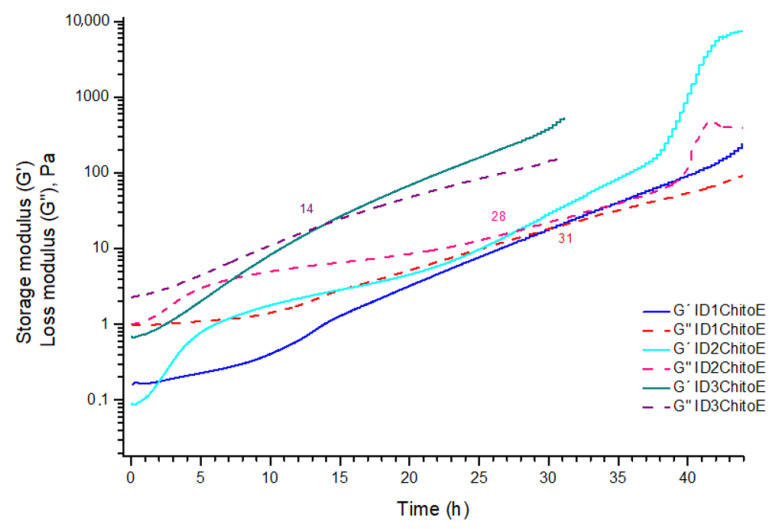
Dependence of the storage modulus (G′) and loss modulus (G″) on time for various polymer solutions highlighting their time-dependent gelation behavior (chitosan solutions made from AcOH).

The crossover point of the storage modulus (G′) and loss modulus (G″) was a key feature observed for all samples (Figure 5). At the beginning of the measurement, the loss modulus exceeded the storage modulus (G″ > G′), indicating predominantly viscous behavior of the chitosan solutions. As solvent evaporation progressed and the polymer network developed, the storage modulus increased continuously and eventually surpassed the loss modulus. The crossover of G′ and G″ is commonly associated with the gelation point, marking the transition from a predominantly liquid-like to a predominantly solid-like response. Depending on the sample composition, this transition occurred after approximately 14, 28, and 31 h, respectively. This transition aligns with dynamic rheology’s description of the gel point, where G′ = G″ is commonly used as a useful indicator to identify the development of a continuous, interconnected polymer network [25]. 

As evidenced by the late crossover of the (G″) and (G′), the ID1ChitoE sample took the longest to reach the gelation point in the rheological investigation. Before this, a primarily viscous behavior was confirmed by G″ dominance over G′. The material exhibits solid-like characteristics after the transition, with G′ exceeding G″. The solution appears to have been fluid until just before gelation, as seen by the notable rise in G″ at 30 h. However, the gelation kinetics of chitosan samples are strongly dependent on their molecular weight (MW) when dissolved in AcOH. Increasing the molecular weight accelerates the gelation process. This trend is evident in samples from ID1ChitoE to ID3ChitoE, which displayed gelation times ranging from 14 to 31 h. The irregular profile of the curves suggests that drying effects during the extended measurement period may have contributed to the observed fluctuations. Low-molecular-weight samples required the longest time to reach the gel point and complete crosslinking, whereas high-molecular-weight samples exhibited significantly faster gelation. 

**Figure 6:**
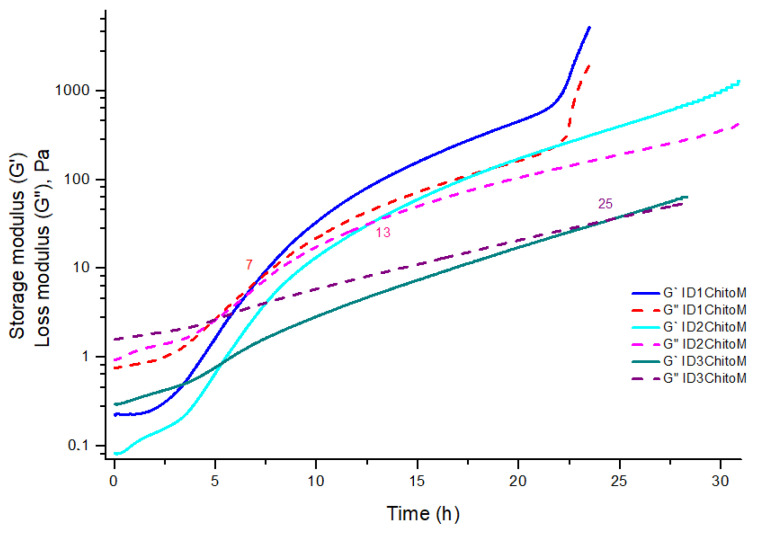
Dependence of storage modulus (G′) and loss modulus (G″) on time for various polymer solutions highlighting their time-dependent gelation behavior (chitosan solutions made from LA).

In contrast to the behavior observed in acetic acid, chitosan dissolved in lactic acid exhibited longer gelation times with increasing molecular weight (ID1ChitoM–ID3ChitoM, Figure 6). This inverse trend is likely related to the stronger polymer–solvent interactions in lactic acid. Due to the presence of both carboxyl and hydroxyl functional groups, lactic acid can form extensive hydrogen-bonding interactions with chitosan, resulting in enhanced chain solvation and increased solution viscosity. These effects become more pronounced at higher molecular weights, where chain entanglement is already significant. The resulting reduction in chain mobility and crosslinker diffusion delays network formation and shifts the gel point to longer times. Consequently, low-molecular-weight chitosan samples gelate more rapidly than their high-molecular-weight counterparts in lactic acid solutions. In particular, compared to the 31 h time recorded for ID1ChitoE, the gelation period of the ID1ChitoM sample was around 7 h. It appears to be a pattern across the lactic acid-based samples that shorter gelation periods are correlated with chitosan of lower MW. The measured gelation points occurred at approximately 7 h (low MW), 13 h (medium MW), and 25 h (high MW).

The rheological results demonstrate that solvent selection governs polymer network formation during gelation. Compared with LA, AcOH promoted denser polymer networks and improved film homogeneity. Consequently, the AcOH-derived films, particularly ID2ChitoE and ID3ChitoE, exhibited the most favorable combination of processability, uniform film formation, and dimensional stability, providing the basis for their selection for subsequent piezoelectric characterization.

### 3.2. Thermal Analysis

#### Thermogravimetric Analysis (TGA)

The thermal stability and thermal degradation profile of the chitosan films were studied by TGA. The investigation provided information on material behavior under controlled heat, particularly the analysis of weight loss associated with the evaporation of moisture and/or of the remaining residual solvent and the onset of decomposition.

**Figure 7:**
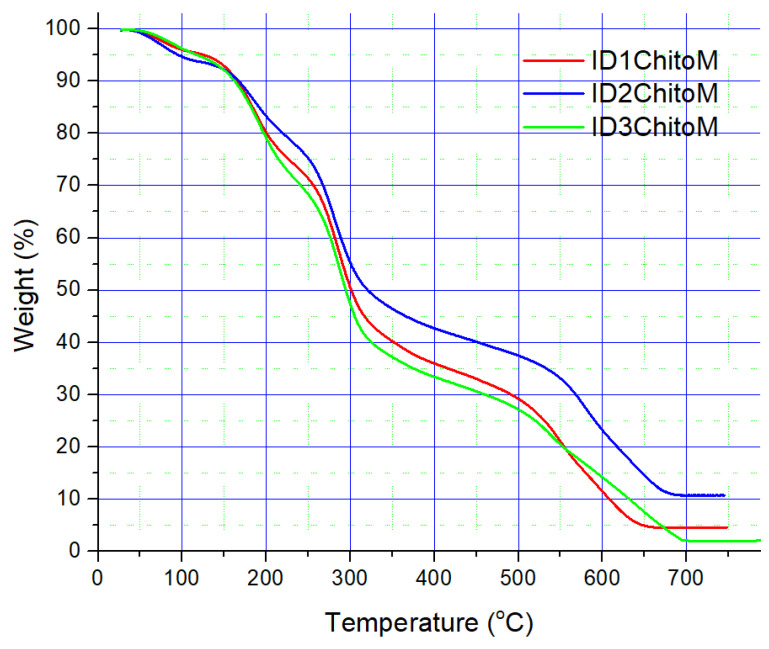
TGA curves of chitosan films (ID1ChitoM, ID2ChitoM, ID3ChitoM) prepared using LA.

The evaporation of moisture and residual solvent causes the initial weight loss in the TGA curve for the LA-based samples (Figure 7). This happens between 50 °C and 150 °C. On average, this weight loss is around 5%. There is a subsequent substantial weight loss between 150 °C and 280 °C, from around 95% to 55% (≈40%). This range corresponds to the boiling point of LA (122 °C), suggesting the possibility of residual acid evaporation and the start of polymer decomposition. However, this region also overlaps with the onset of the thermal degradation of chitosan, which generally begins above approximately 200 °C and involves depolymerization, cleavage of glycosidic (C–O–C) linkages, and degradation of the polysaccharide backbone. The slope of the curve varies from sample to sample above 280 °C, which might be caused by changes in film thickness and MW of the utilized chitosan. Degradation behavior and heat transport are influenced by these characteristics. Additional factors that could be responsible for the observed variability include the sample preparation technique, including the quantity, dimensions, and configuration of the film fragments inside the crucible. Laboratory experiments have verified that the LA-based films visually turn brown over 190 °C. Under regulated heating conditions, an infrared thermometer recorded a surface temperature of around 199.4 °C, confirming the early degradation and thermal instability seen at this point.

**Figure 8:**
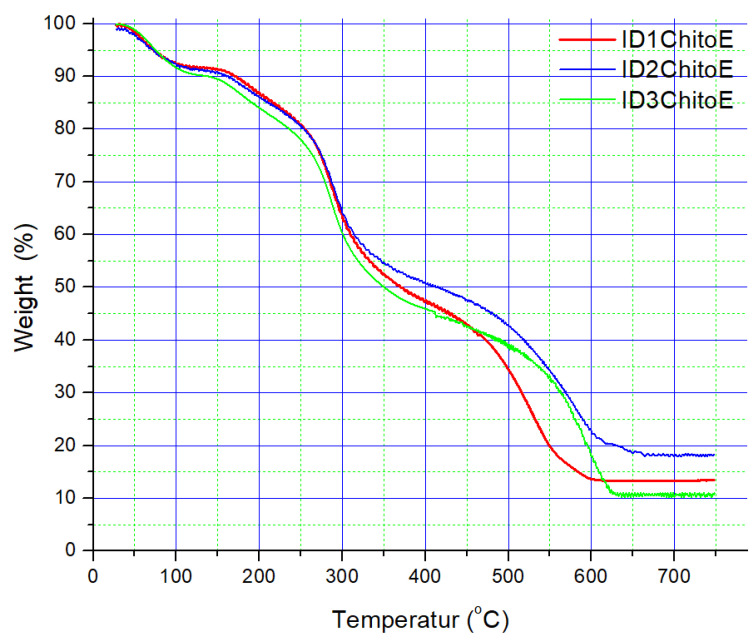
TGA curves of chitosan films (ID1ChitoE, ID2ChitoE, ID3ChitoE) prepared using AcOH.

The evaporation of moisture results in an initial weight loss at around 100 °C of roughly 10% for AcOH-based samples according to the TGA plot (Figure 8). There are two distinct steps of weight loss, with an intermediate step that contributes about 25% of the overall weight decrease and occurs between 150 °C and 260 °C. The evaporation of remaining AcOH (bp 118 °C) may be related to this intermediate zone. But compared to the films made with LA (Figure 5), the impact is less noticeable, indicating that AcOH evaporates completely during the drying process, leaving fewer solvent residues.

The thermal breakdown starts at 320–330 °C. This is evidenced by the subsequent weight loss, which goes from about 75% to 45% (≈30%). These interpretations align with findings presented by Ehrenstein et al., who elaborate extensively on thermal degradation behavior in polymer systems [26]. Visual comparisons also confirm this observation: Figure 7 and Figure 8 show that films formed from LA are noticeably more elastic and flexible, whereas those made from AcOH are noticeably stiffer. These physical variations highlight how the type of solvent affects the final chitosan films’ mechanical and thermal characteristics.

The thermal degradation process was validated experimentally by heating chitosan films based on AcOH in aluminum dishes. A thermal infrared device was employed to record a surface temperature of around 193.6 °C, confirming the films’ visible browning and heat degradation above 190 °C. TGA revealed only minor differences in thermal stability between the AcOH- and LA-derived films, indicating that solvent selection primarily affected the polymer structure and film morphology rather than the intrinsic thermal stability of the materials.

### 3.3. Thermo-Optical Measurements by m-Line Spectroscopy

The purpose of this study was to determine the glass transition temperature (T_g_) and thermo-optic coefficient (TOC). To avoid possible effects of residual solvent evaporation, the films were preliminarily dried in a vacuum oven. The technique allows for evaluating the thermal response, rigidity, and elasticity of the films. Analyzing these characteristics was essential for comprehending the materials’ potential performance in actual applications, especially with regard to their thermal expansion and mechanical flexibility. 

**Figure 9:**
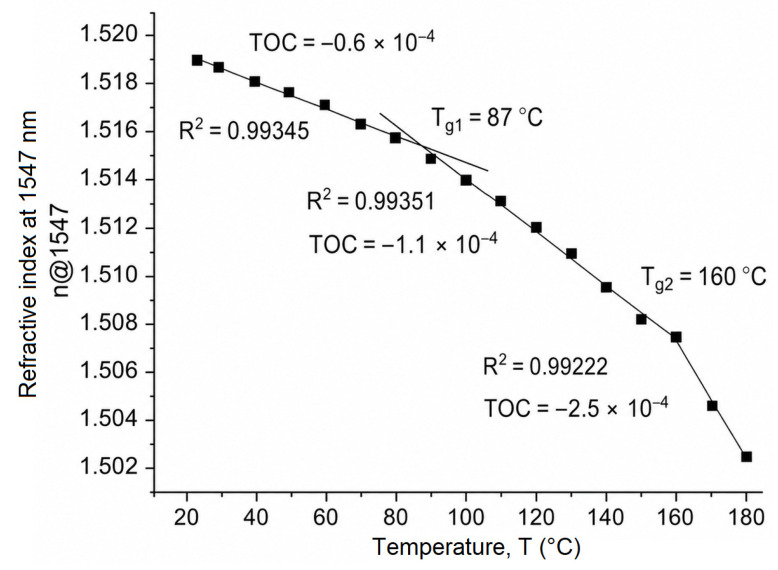
Dependence of refractive index at 1547 nm on the temperature for the chitosan film ID1ChitoM (26 µm thick) pressed to the Si waver (single mode measurement).

A representative temperature-dependent refractive index measurement is shown in Figure 9. The refractive index decreased continuously with increasing temperature and exhibited two distinct linear regions. The glass transition temperature was determined from the intersection of the corresponding linear fits and was found to be approximately 152 °C. The excellent linearity of both regions (R^2^ = 0.998 and 0.999, respectively) confirms the reliability of the analysis. Below Tg, the thermo-optic coefficient was −1.9 × 10^−4^ K^−1^, while above Tg it increased in magnitude to −3.2 × 10^−4^ K^−1^. The pronounced change in TOC reflects the onset of increased segmental mobility of the polymer chains above the glass transition temperature. Such behavior is characteristic of amorphous thermoplastic materials. Furthermore, the relatively high magnitude of the TOC below Tg suggests a certain degree of molecular mobility already at room temperature, which may be attributed to the plasticizing effect of residual lactic acid remaining in the film.

Table 2 summarizes the TOC values measured below and above T_g_. The TOC is closely related to thermal expansion and molecular mobility and therefore provides valuable information about the stiffness and elasticity of polymer films [27]. 

In general, lower TOC values indicate reduced thermal sensitivity, lower thermal expansion, and a more rigid polymer structure, whereas higher TOC values are associated with increased chain mobility and softer materials. The lowest TOC values were observed for ID1ChitoE and ID2ChitoE, suggesting relatively stiff polymer networks with restricted molecular mobility. In contrast, ID1ChitoM exhibited the highest TOC values both below and above Tg, indicating enhanced thermal responsiveness and a softer polymer structure. This observation agrees with the visual and morphological characterization of the films and supports the interpretation that residual lactic acid acts as a plasticizer.

Films based on LA (ID1ChitoM and ID2ChitoM) exhibit significantly higher thermo-optic coefficients (TOC) than the corresponding AcOH-based films, both below and above T_g_. Since the TOC reflects changes in refractive index with temperature, higher TOC values indicate a stronger temperature dependence of the polymer structure and a greater sensitivity of molecular packing to thermal expansion. The pronounced increase in TOC above T_g_ is consistent with the onset of cooperative segmental motion of the polymer chains, resulting in increased free volume and enhanced molecular mobility. This behavior is characteristic of materials with a more pronounced viscoelastic response and suggests that the LA-based films possess a less rigid polymer network than the AcOH-based films. In contrast, the lower TOC values observed for the AcOH-based samples indicate more restricted chain mobility and a stiffer molecular structure. Therefore, the differences in thermo-optical behavior are more likely associated with variations in molecular structure and polymer–acid interactions. Overall, the results indicate that the type of acid used during film preparation has a greater influence on the thermal and thermo-optical properties of the films than the molecular weight of chitosan. Lactic acid promotes higher molecular mobility and stronger thermo-optical responses, whereas acetic acid produces films with lower TOC values and a more rigid molecular structure.

**Figure 10:**
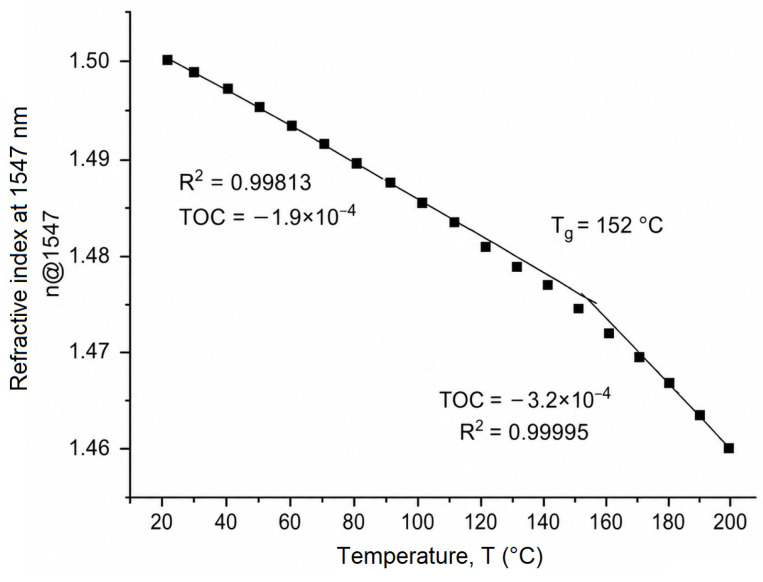
Dependence of refractive index at 1547 nm on temperature for the chitosan film ID2ChitoE (27 µm thick) pressed to the Si waver (single mode measurement).

Since crystalline and amorphous domains respond differently to temperature changes, the coexistence of both phases in semi-crystalline polymers can lead to multiple thermal transitions. This phenomenon is well established in polymer science. Crystalline polymers consist of complex structural elements combining crystalline and amorphous domains at very small length scales, which may result in several phase transitions upon heating [28]. Furthermore, heterogeneous polymer systems containing both crystalline and amorphous regions frequently exhibit multiple thermal transitions due to differences in morphology and crystallization behavior [27]. The dual transitions observed in the n(T) curve of ID2ChitoE (Figure 10), at approximately 87 and 160 °C, are consistent with the presence of both amorphous and crystalline domains contributing to the thermal behavior of the material. The lower transition can be attributed to the glass transition of the amorphous phase, whereas the higher transition may be associated with relaxation processes occurring in more ordered regions. Therefore, the dual thermal transitions observed for ID2ChitoE, indicating the coexistence of amorphous and crystalline domains, suggest that this material is a promising candidate for the development of chitosan-based materials with potential piezoelectric properties and, next, for functional integration into composite materials. 

### 3.4. FTIR Analysis 

**Figure 11:**
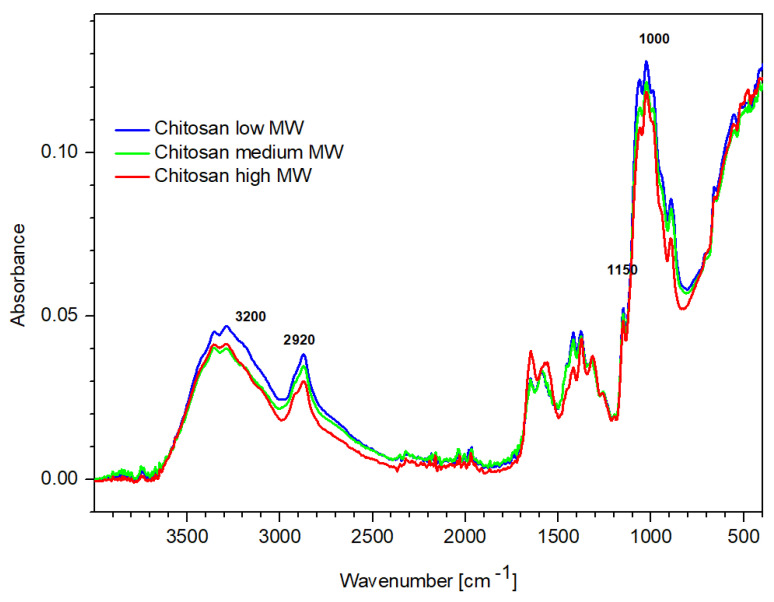
FTIR spectra overlay of various chitosan powders.

FTIR spectra of pure chitosan with different molecular weights (low, medium, and high MW) exhibit the characteristic absorption bands of chitosan, including a broad band at 3200–3500 cm^−1^ attributed to O–H and N–H stretching vibrations, bands around 2920–2870 cm^−1^ corresponding to C–H stretching vibrations, and the characteristic polysaccharide bands between 1150 and 1000 cm^−1^ associated with C–O–C and C–O stretching vibrations (Figure 11).

The spectra of the three chitosans with different molecular weights are highly similar, indicating that molecular weight has little influence on the chemical structure of chitosan. Only slight differences in absorbance intensity are observed, particularly in the hydroxyl and amino stretching region.

**Figure 12:**
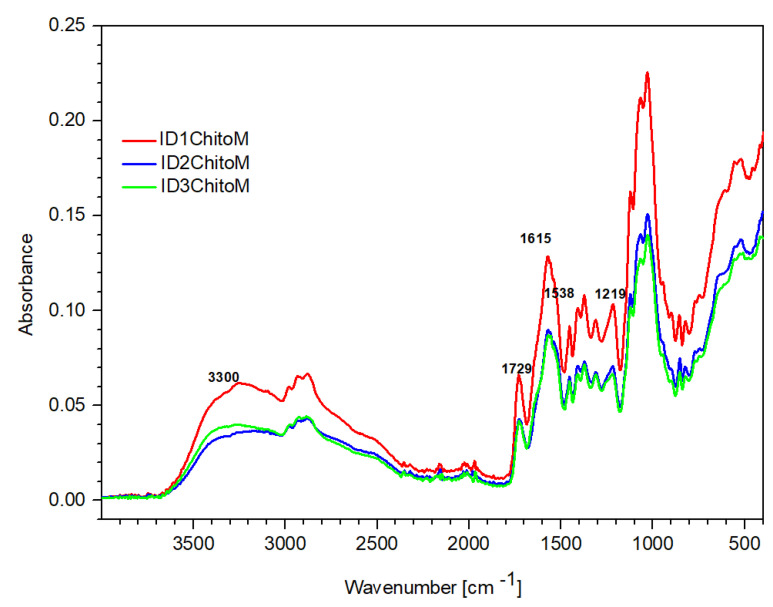
FTIR spectra overlay of chitosan films prepared from LA.

**Figure 13:**
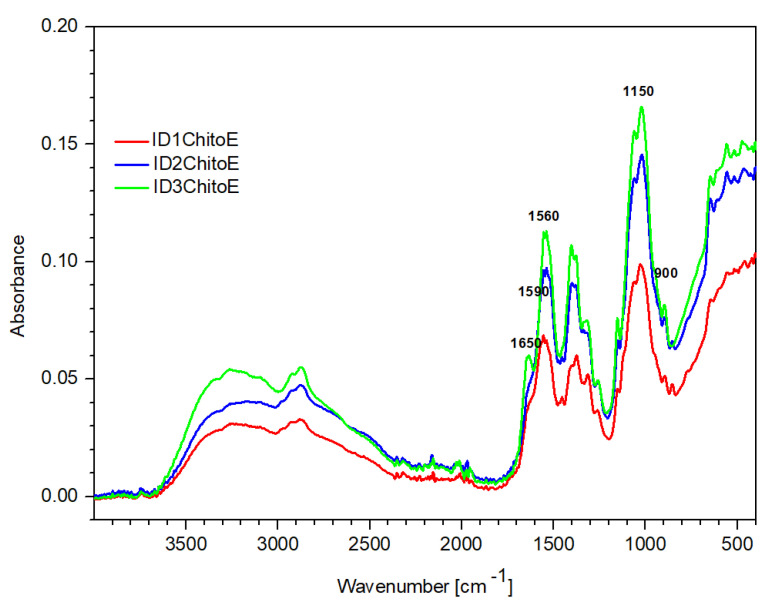
FTIR overlay spectra of chitosan films prepared from AcOH.

FTIR spectra of chitosan powders (Figure 11) and films prepared from LA (Figure 12) and AcOH (Figure 13) reveal characteristic absorption bands associated with chitosan’s molecular structure. A broad band in the 3000–3500 cm^−1^ region corresponds to overlapping O–H and N–H infrared-active stretching vibrations. The existence of carbonyl groups is indicated by the C=O stretching band around 1650 cm^−1^. Absorptions at 1560–1590 cm^−1^ are allocated to N–H bending vibrations of amines and secondary amides. The C–O–C stretching of the saccharide ring appears near 1150 cm^−1^, while the spectrum between 900 and 1100 cm^−1^ displays bands characteristic of polysaccharide structures.

In addition to the peaks associated with chitosan, the FTIR spectrum analysis of the film prepared from LA (Figure 12) also shows some carbonic acid-specific peaks, such as C=O at 1729 cm^−1^, which is linked to the lactate, with C-O asymmetric at 1219 cm^−1^. Furthermore, for both films, IDChitoE and IDChitoM, peaks related to the protonation of amines can be observed in the ranges of 1538 cm^−1^ and 1615 cm^−1^ [29]. The shift of the characteristic chitosan peaks may suggest interactions involving the amine radical of chitosan and the carbonyl radicals of the acids. The spectra of chitosan dissolved in acetic acid display similar but distinct changes. As observed for the lactic acid system, the broad O–H/N–H stretching band increases in intensity due to hydrogen bonding and protonation effects [30]. These results confirm successful acid–chitosan interactions and the formation of protonated chitosan salts in both systems.

Based on the comprehensive characterization presented in this study, the AcOH-derived films ID2ChitoE and ID3ChitoE were selected for subsequent piezoelectric characterization because they demonstrated the most favorable combination of processability, structural homogeneity, dimensional stability, and thermomechanical performance, making them the most suitable candidates for functional integration into composite materials.

### 3.5. Electromechanical Characterization by Force Excitation

The focus of this characterization is to examine the potential of these films for sensing pressures or pressure changes. Measuring these qualities is essential for evaluation of their appropriateness in composite materials and functional applications requiring sensor characteristics.

As described above, the force excitation (*F*_pp_) and charge displacement response (*Q*_pp_) for every excitation cycle were measured. Considering the great sensitivity of the charge detection towards external disturbances, a total of nine separate measurements were made per force amplitude in order to ensure data reliability and reduce the impact of electrical interference. The ratio of charge to force (Q_pp_/F_pp_) was used to compute the sensor activity for each measurement, and the median of the nine values was determined as the final result. This method was implemented to reduce the impact of statistical outliers brought on by interferences. In order to examine whether electrical poling induces or enhances the sensor activity, some samples were exposed to an electric field of 20 MV/m, and other samples to 60 MV/m.

The measurements revealed that only ID3ChitoE exhibited detectable sensor activity after electrical pretreatment. All other samples showed signals only within the experimental noise level. This suggests that the structural and molecular organization present in ID3ChitoE is more favorable for sensor purposes than in the other investigated chitosan films.

**Figure 14:**
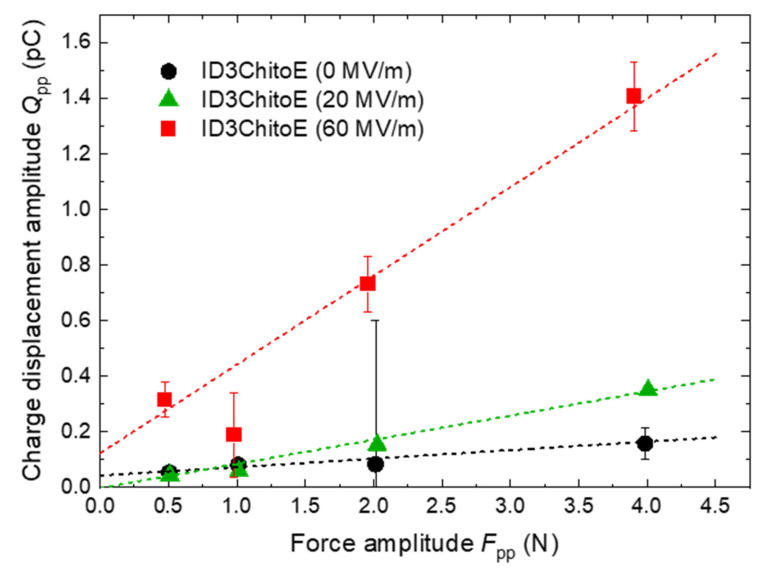
Measured charge displacement amplitude as a function of applied force amplitude in order to characterize the sensor function of sample ID3ChitoE.

The charge displacement amplitudes measured on various electrically pretreated ID3ChitoE samples as a function of different applied force amplitudes are presented in Figure 14. Here, the prepared films, which have not received any electrical pretreatment, show only extremely weak charge displacement amplitudes even at higher force amplitudes. After pretreatment with an electric field of 20 MV/m, the charge displacement amplitude increases slightly but still remains in the range of very low absolute values. Electrical pretreatment with 60 MV/m significantly increases the charge displacement amplitude and leads to a verifiable sensor function; however, it still has low values for the coefficient Qpp divided by Fpp.

Although relatively moderate, the observed trend suggests a positive relationship between mechanical excitation and electrical pretreatment towards electrical output.

### 3.6. Study Novelty and Limitations

The novelty of this study lies in the systematic evaluation of commercially available chitosan grades processed using two different solvent systems (acetic acid and lactic acid) and the correlation of their rheological, thermo-optical, thermal, structural, and electromechanical properties within a unified experimental framework. Rather than investigating an isolated material parameter, the study aims to identify practically processable chitosan formulations suitable for sustainable functional integration into composite materials. Several limitations should be acknowledged. The commercial chitosan grades differed not only in molecular weight but also in degree of deacetylation, and therefore the observed differences reflect the combined influence of these material characteristics. Furthermore, the amount of undissolved material removed during filtration was not quantified. The rheological measurements intentionally incorporated solvent evaporation to simulate film formation, meaning that the measured crossover times represent comparative indicators of network development rather than intrinsic gelation kinetics. 

## 4. Conclusions and Outlook

In this work, chitosan-based biopolymer films were successfully prepared using a simple solution-casting process, and the influence of solvent type (acetic acid and lactic acid) as well as chitosan molecular weight on their structural, thermal, rheological, thermo-optical, and mechanoelectrical properties was systematically investigated. The developed films were transparent, homogeneous, and mechanically stable, demonstrating their suitability as sustainable materials for future functional integration into polymer composite systems.

The rheological investigations revealed that both solvent type and molecular weight strongly influence polymer network formation and gelation behavior. Acetic acid promoted the formation of denser and more rigid polymer networks, whereas lactic acid produced films with higher molecular mobility and greater elasticity. Increasing the molecular weight of chitosan resulted in higher solution viscosity and more difficult dissolution due to enhanced chain entanglement. High-molecular-weight chitosan required longer dissolution times and, in some cases, filtration prior to casting. Despite these processing challenges, the resulting films exhibited improved homogeneity, dimensional stability, and mechanical integrity, indicating that higher molecular weight is advantageous for producing robust and high-quality films. In particular, the medium- and high-molecular-weight chitosan formulations prepared with acetic acid provided the most favorable balance between processability, film quality, and structural stability.

TGA demonstrated that the thermal behavior of the films was influenced by the solvent system used during preparation. The acetic acid- and lactic acid-derived films exhibited distinct degradation profiles, particularly within the intermediate temperature range, reflecting differences in residual solvent interactions, polymer morphology, and the onset of thermal degradation. For the lactic acid-based films, the observed mass-loss region is interpreted as the result of overlapping processes, including the removal of strongly retained residual solvent and the initial degradation of the chitosan backbone. In contrast, the AcOH-based films show lower TOC values and a smaller change above T_g_, indicating a stiffer polymer network with more restricted molecular motion. Their TGA behavior is broadly similar to the M-series, suggesting that the difference in TOC is not mainly due to thermal degradation behavior, but rather to differences in polymer structure.

A mechanoelectric sensor functionality was demonstrated in at least one modification of the chitosan-based samples, especially on films prepared from ID3ChitoE. This functionality is still very low in the films as prepared but seems to increase with the degree of electrical pretreatment. However, this shows that further treatment of the samples could in principle make it possible to optimize sensor functionality. The underlying structural or charge-related modifications caused by the electrical pretreatment are the subject of further investigations.

Future research may explore techniques to improve the electro-specific characteristics of chitosan-derived biopolymer films. This involves the analysis of alignment methods, such as electric field-assisted casting, which might enhance molecular orientation, with the integration of functional nanomaterials or polar additives to facilitate dipolar interactions. Increased focus should be directed towards crosslinking strategies such as enzymatic, ionic, or photo-initiated methods that preserve film flexibility while enhancing the molecular alignment within films. Moreover, optimizing the film casting process to ensure uniform thickness and reduce optical scattering is crucial for improving the consistency and quality of thermal and optical analyses. The findings provide a strong foundation for further material optimization and highlight the importance of molecular structure and processing conditions in determining electroactive performance. Future investigations focusing on long-term stability, biodegradability, and mechanical durability under cyclic loading will further support the development of these sustainable materials for applications in sensors. Moreover, analyzing long-term stability, biodegradability, and mechanical performance under cyclic stress will be beneficial for investigating the possibility of these materials in sensor, packaging, and biomedical applications.

## Figures and Tables

**Table 1:** Formulations of chitosan films prepared.

Sample Name	Chitosan Type	Chitosan Amount 1 (wt%)	AcOH 1 (wt% Solution)	LA 1 (wt% Solution)	Weight of the Dry Sample/mg	Average Thickness/µm
ID1ChitoE	Low MW	1	yes	-	900	26
ID1ChitoM	Low MW	1	-	yes	1167	14
ID2ChitoE	Medium MW	1	yes	-	677	40
ID2ChitoM	Medium MW	1	-	yes	1113	27
ID3ChitoE	High MW	1	yes	-	780	43
ID3ChitoM	High MW	1	-	yes	776	27

**Table 2:** Results of m-line temperature-dependent measurements.

Sample Name	TOC Before T_g_ (×10^−4^ K^−1^)	TOC After T_g_ (×10^−4^ K^−1^)	T_g_ (°C)	Thickness (µm)	Interpretation
ID1ChitoM	−1.9	−3.2	142	26	highest TOC values indicate enhanced chain mobility and strong plasticization
ID1ChitoE	−0.6	−1.0	95	14	lowest TOC values suggest restricted segmental motion
ID2ChitoM	−1.4	−1.9	80	40	intermediate TOC values consistent with conventional thermoplastic behavior
ID2ChitoE	−0.5	−1.1	87 and 160	27	reduced thermo-optic response indicating limited molecular mobility
ID3ChitoM	−1.5	/	/	43	absence of detectable T_g_ below 200 °C suggests a thermally stable amorphous phase
ID3ChitoE	−1.15	−2.0	62	27	intermediate thermo-optic response with moderate chain mobility

## Data Availability

The original contributions presented in this study are included in the article. Further inquiries can be directed to the corresponding authors.
